# Development of a Bead-Based Multiplex Assay for Use in Multianalyte Screening and Surveillance of HIV, Viral Hepatitis, Syphilis, and Herpes

**DOI:** 10.1128/jcm.02348-21

**Published:** 2022-04-07

**Authors:** Ernest L. Yufenyuy, Shanmugam Vedapuri, Amy Zheng, Gretchen Cooley, Damien Danavall, Shukla Mayur, Maja Kodani, Cheng Chen, Ye Tun, Yetunde F. Fakile, Diana Martin, Saleem Kamili, Kevin Karem, Bharat S. Parekh

**Affiliations:** a Division of Global HIV and TB, Centers for Disease Control and Prevention, Atlanta, Georgia, USA; b Public Health Institute/Centers for Disease Control and Prevention Global Health Fellowship Program, Oakland, California, USA; c Division of Parasitic Diseases and Malaria, Centers for Disease Control and Prevention, Atlanta, Georgia, USA; d Division of STD Prevention, Centers for Disease Control and Prevention, Atlanta, Georgia, USA; e Division of Viral Hepatitis, Centers for Disease Control and Prevention, Atlanta, Georgia, USA; f Center for Global Health, Centers for Disease Control and Prevention, Atlanta, Georgia, USA; Mayo Clinic

**Keywords:** multipathogen, multiplex, blood-borne

## Abstract

Diagnostic assays that can simultaneously determine the presence of infection with multiple pathogens are key for diagnosis and surveillance. Current multiplex diagnostic assays are complex and often have limited availability. We developed a simple, multianalyte, pathogen detection assay for screening and serosurveillance using the Luminex Magpix platform that is high throughput and can be helpful in monitoring multiple diseases. The Luminex bead-based 10-plex immunoassay for the detection of HIV-1, HIV-2, Treponema pallidum, hepatitis B virus (HBV), hepatitis C virus (HCV), herpes simplex virus 1 (HSV-1), and HSV-2 infections was accomplished by coupling beads with specific antigens to detect IgG antibodies in plasma or serum samples. Each coupled antigen was systematically optimized, and the performance was evaluated using a panel of well-characterized specimens (*n* = 417) that contained antibodies to HIV-1, HIV-2, T. pallidum, HBV, HCV, HSV-1, and HSV-2. The multiplex assay had a sensitivity of 92.2% (95% Clopper-Pearson confidence interval [CI], 90.2 to 94.0%) and a specificity of 98.1% (95% CI, 97.6 to 98.7%). The sensitivities and specificities for disease-specific biomarker detection ranged from 68.7 to 100% and 95.6 to 100%, respectively. The results showed that the 10-plex immunoassay had an overall agreement of 96.7% (95% CI, 96.7 to 97.3%) with reference tests and a corresponding kappa value of 0.91 (95% CI, 0.90 to 0.93). Kappa values for the individual pathogens ranged from 0.69 to 1.00. The assay is robust and allows the simultaneous detection of antibodies to multiple antigens using a small sample volume in a high-throughput format. This assay has the potential to simplify disease surveillance by providing an alternative to expensive and highly specialized individual tests.

## INTRODUCTION

Global efforts to control the transmission of infectious diseases are critically important (1–4), but the absence of inexpensive diagnostic tools in the field for robust disease surveillance has slowed down progress, especially for resource-limited countries. Most recent intervention efforts have focused on mother-to-child transmissions (MTCTs), where prevailing recommendations on MTCT revolve around comprehensive and improved identification and management of infections in an integrated surveillance system (1, 5, 6). However, disease-specific biomarker surveillance activities are not integrated in most low- and middle-income countries because of the enormous cost associated with current disease detection and survey methods, which largely use single or dual biomarkers for detection. Viral hepatitis, Treponema pallidum, and herpes simplex virus (HSV) screenings have been evaluated for potential integration into HIV screening programs in many countries (7–11). However, success has been limited because of the complexities and costs associated with integrating disease screening. Utilizing HIV prevention programs as a platform for integrated disease surveillance could prove beneficial given that significant progress has already been observed in HIV surveillance that has led to informed public health decisions with great impact. These have resulted in a worldwide reduction in HIV incidence and reductions in MTCT of HIV, AIDS cases, and AIDS-related deaths (8, 12–14). An example of an informed public health decision is the adoption of Option B+, a WHO recommendation, which affords HIV-positive expectant mothers immediate and lifelong HIV treatment regardless of CD4 counts to prevent the vertical transmission of HIV (15). This has resulted in a drastic reduction in MTCT of HIV as a direct outcome of planned public health activity. However, not all pathogens known to transmit from mother to child have received the same attention as HIV, and the lack of surveillance data on the prevalence and incidence of these pathogens has slowed progress in controlling or eliminating the diseases that they cause. For example, viral hepatitis has been termed the “silent killer” because despite its significant burden on society, it has been largely ignored. However, the prevalence and incidence of the disease caused by hepatitis viruses, especially hepatitis B virus (HBV) and hepatitis C virus (HCV), remain relatively high. Viral hepatitis causes almost 1.5 million deaths worldwide and was ignored as a health priority until recently (3). Innovative approaches for the surveillance of MTCT and an eventual response strategy are needed to support low-income countries in developing an integrated surveillance system.

Early diagnosis of infections and treatment improve long-term outcomes and prevent vertical transmissions of infectious diseases (1, 16, 17). Rapid tests with single and multiple biomarkers are gaining popularity in low-income countries. For example, the dual rapid HIV and T. pallidum diagnostic test has been utilized in integrated screening systems (14, 18). The implementation and uptake of such rapid tests have been limited and slow because only a couple of biomarkers can be tested with various sensitivities, reading test results can be subjective, and the tests cannot be applied in high-throughput formats and are often expensive for large surveys and routine use. All of these make it difficult for low-income countries to successfully integrate MTCT surveillance.

Laboratory-developed assays capable of simultaneously detecting multiple pathogens using a single specimen can support the more cost-effective implementation of integrated disease surveillance in resource-constrained settings. Ideally, such assays should be minimally invasive for the person tested (that is, require a very small sample volume), easy to collect for the health provider, and simple to use. The use of the bead-based multiplex assay is gaining traction in diagnosis because of the ease, high throughput, and minimal sample/reagent volume requirements (19–22). However, a platform with high multiplexing capability has not been widely applied in the simultaneous detection of multiple pathogens associated with vertical transmission.

The Luminex multiplex platform (Luminex Biotechnology, Austin, TX, USA) is a magnetic bead-based technology that allows the simultaneous measurement of multiple analytes in a single test. A Luminex-based system for HIV prevalence and incidence (22), yaws (23), malaria (24), viral hepatitis (25), T. pallidum (26), and several other diseases has been developed (20). In this study, we combined 7 new analytes together with 3 HIV-1 and HIV-2 analytes to develop an integrated biomarker detection platform using highly specific antigens. Our study demonstrates that the 10-plex system has the potential to be useful in high-throughput assay formats and may help in the surveillance of MTCT infections. However, the current work represents a pilot proof-of-concept study involving precharacterized samples with an origin not necessarily from mothers or infant children.

## MATERIALS AND METHODS

### Specimens.

A total of 6 plasma/serum panels were used in this study. The initial 5 panels (panel 1 to panel 5) were small panels consisting of 8 to 16 specimens used for the optimization of key assay parameters (antigen type and concentration for coupling and cutoffs for positive and negative results) for each biomarker (Table 1). The final panel (*n* = 417) was put together to evaluate the performance of the multiplex assay combining all biomarkers in a single assay.

**TABLE 1:** Specimens used for development^*a*^

Specimen panel	No. of specimens	Pathogen(s)	Standard/commercial assay(s)	Purpose
Panel 1	13	HIV-1/2	EIA/Western/Multispot	Optimization
Panel 2	8	Syphilis	RPR, TP-PA, Trep-Sure	Optimization
Panel 3	15	Hepatitis C	Vitros 3600 platform	Optimization
Panel 4	13	Hepatitis B	Vitros 3600 platform	Optimization
Panel 5	16	HSV-1 and -2	Final Focus	Optimization
PEP	417	All	All	Performance/evaluation

a Panel specimens were well characterized using the assays indicated.

Panel 1 was a 13-member panel that contained antibodies to HIV-1 (*n* = 4) and HIV-2 (*n* = 7). Two of the specimens in the panel did not have antibodies to either HIV-1 or HIV-2. Panel 2 consisted of 8 specimens. Four were antibody positive for T. pallidum, while 4 were antibody negative for T. pallidum.

Panel 3 consisted of 7 anti-HCV antibody-positive specimens and 8 anti-HCV-negative specimens.

Panel 4 had 7 specimens positive for total antibodies to hepatitis B core antigen (total anti-HBc) and 6 total anti-HBc-negative specimens.

Panel 5 consisted of 7 HSV-1-positive specimens and 9 negative specimens. This 16-member panel also had 7 HSV-2-positive specimens and 9 HSV-2-negative specimens. Only 1 specimen was positive for both HSV-1 and HSV-2.

The performance evaluation panel (PEP) was made up of 417 specimens (HIV-1 and HIV-2, *n* = 85; T. pallidum, *n* = 85; HBV and HCV, *n* = 85; HSV-1 and HSV-2, *n* = 85; supplemental panel, *n* = 77) and was used to evaluate the performance of the 10-plex assay parameters. Note that the performance evaluation panel contained both positive and negative specimens for each of the pathogens in the panels.

### Characterization of panels. (i) HIV.

The HIV 85-member subpanel consisted of well-characterized HIV-1 (*n* = 35)-, HIV-2 (*n* = 3)-, and HIV-1/2 (*n* = 2)-positive specimens and negative specimens (*n* = 45). These were characterized by the 3rd-generation Genetic Systems HIV-1/2 Plus O enzyme immunoassay (EIA) (Bio-Rad Laboratories, Hercules, CA) and the Cambridge Biotech HIV Western blot assay (Maxim Biomedical, Rockville, MD) using a standard algorithm. Serotyping was achieved with the Multispot HIV-1/2 assay and the Geenius HIV-1/2 supplemental assay, which detects and differentiates HIV-1 and HIV-2 antibodies (Bio-Rad). These specimens serve as reference specimens in the HIV serology reference laboratory at the CDC and were acquired from Boca Biolistics Inc. (Pompano Beach, FL). The same mode of characterization was used for panel 1 specimens.

### (ii) Syphilis.

The T. pallidum 85-member subpanel was prepared from residual specimens collected from the Georgia Public Health Laboratory (Atlanta, GA), which were deidentified and unlinked. All specimens were tested by the rapid plasma reagin (RPR) (Arlington Scientific, UT), T. pallidum particle agglutination (TP-PA) (Fujirebio, Japan), and Trep-Sure enzyme immunoassay (EIA) (Trinity Biotech, Ireland) in the Laboratory Reference and Research Branch (CDC, Atlanta, GA).

Specimens were categorized based on their reactivity as described by the manufacturers. Thirty-two specimens were reactive by RPR, TP-PA, and Trep-Sure EIA and were grouped as suggestive active infection (SAI). Twenty specimens were nonreactive by all tests and were grouped as suggestive nonreactive (SN), while 10 specimens were nonreactive by RPR and reactive by TP-PA and Trep-Sure EIA and were grouped as suggestive past infection (SPI) or incubating early infection (IEI). Nineteen specimens were obtained that had reactive RPR but nonreactive TP-PA and Trep-sure EIA results and were classified as biological false positive (BFP), and the remaining four specimens were grouped into the indeterminate category based on their inconclusive or discrepant results among the different tests used. The same mode of characterization was used for panel 2 specimens.

### (iii) Viral hepatitis.

The viral hepatitis 85-member subpanel consisted of 32 anti-HCV-positive plasma specimens, 10 total anti-HBc-positive plasma specimens, and 10 anti-HCV/total anti-HBc-positive specimens, tested on the Vitros 3600 platform (Ortho Clinical Diagnostics, Germany). These specimens were obtained from a U.S. plasma donor center that rejected them due to positivity for hepatitis markers of infection. The subpanel also included 33 plasma specimens negative for anti-HCV and total anti-HBc. The same mode of characterization was used for panel 3 and 4 specimens.

### (iv) Herpes simplex virus.

The HSV 85-member subpanel was provided by the Herpesvirus Laboratory Branch, Division of Viral Diseases, CDC. This panel consisted of a well-characterized and evenly proportioned mix of sera that tested HSV-1 positive (HSV-1^+^)/HSV-2 negative (HSV-2^−^), HSV-2^+^/HSV-1^−^, HSV-1^+^/HSV-2^−^, and HSV-1^−^/HSV-2^−^. The panel was characterized by enzyme-linked immunosorbent assay (ELISA)-based Captia HSV-1 and HSV-2 type-specific IgG assays (Trinity Biotech, Jamestown, NY) and confirmed by testing with a HerpeSelect 1 or 2 IgG ELISA (Focus Diagnostics, Cypress, CA) for the qualitative detection of type-specific IgG antibodies to HSV-1 and HSV-2. Twenty-four out of 85 specimens tested were dually positive for HSV-1 and HSV-2, 22 specimens were positive for HSV-1 only, 19 specimens were positive for HSV-2 only, and 20 specimens were negative for both HSV-1 and HSV-2. The same mode of characterization was used for panel 5 specimens.

### (v) Supplemental panel.

The 77-member supplemental panel was commercially acquired from the Medical Research Networx (MRN) group (West Wareham, MA). This panel was procured for a different purpose but was used to supplement the number of specimens in the evaluation panel. The supplemental panel served more like blinded specimens that were added to the performance evaluation panel, thereby mimicking specimens as they would be encountered and tested in the real world. The supplemental panel also added to the total number of specimens characterized and used for assay performance evaluation.

### Coupling of antigens to beads.

Each antigen was coupled to a specific bead region, and the coupling reaction and assay were performed as described previously (22). The optimal concentration for each antigen (Table 2) to detect IgG antibodies was determined, and a single batch of beads was coupled for all experiments to avoid lot-to-lot variability in bead couplings.

**TABLE 2:** Antigens used for development^*a*^

Antigen	Pathogen	Optimum antigen concn (μg)	Purpose
p24-gp41 protein	HIV-1	1	HIV-1 diagnosis
rIDR-M protein	HIV-2	0.04	HIV-1/2 diagnosis
HIV-2 IDR peptide	HIV-2	10	HIV-2 serotyping
p17-βgal	T. pallidum	4.5	Syphilis diagnosis
TmpA-βgal	T. pallidum	1.5	Syphilis diagnosis
HCV-239	HCV	1	Hepatitis C diagnosis
HCV-11	HCV	0.5	Hepatitis C diagnosis
HBV-270a	HBV	10	Hepatitis B diagnosis
HSV-1gG	HSV-1	1	HSV-1 diagnosis
HSV-2gG	HSV-2	1	HSV-2 diagnosis

a Critical assay parameters show the antigen used and the optimal antigen concentration for coupling, which was experimentally determined.

### (i) HIV antigens.

The p24-gp41 (Bioprocess Inc., Australia) antigen for HIV-1 diagnosis and the HIV2 gp36 immunodominant region (IDR) peptide (CDC Protein Core Facility, Atlanta, GA) for HIV-2 serotyping were coupled on bead regions 12 and 14, respectively. The rIDR-M is the recombinant immunodominat region of group M viruses gp41 (rIDR-M) antigen, which was coupled on bead region 13, was used to guide the classification of HIV-2 and HIV-1/2 dual infections. These antigens have been successfully used for the diagnosis of HIV-1, the serotyping of HIV-2, and the separation of recent from long-term infections (22, 27). However, and by design, the rIDR-M protein was specifically used in this format for a different purpose: to distinguish between dual HIV infections and HIV-2-specific infections.

### (ii) T. pallidum antigens.

Two different antigens derived from T. pallidum (the pathogen causing syphilis) were used: full-length recombinant TmpA-βgal (ViroGen, Watertown, MA) and recombinant p17-βgal (ViroGen, Watertown, MA). Both antigens have been previously used on the Luminex platform for the detection of treponemal antigen-specific antibodies associated with yaws (23).

### (iii) HCV and HBV antigens.

The HCV antigens (HCV-11 and HCV-239) were purchased from ProSpec Protein Specialists (TechnoGene Ltd., Ness Ziona, Israel). HCV-11 is a recombinant HCV core antigen that was supplied in a lyophilized form, while HCV-239 was supplied in phosphate-buffered saline (PBS) with 25 mM arginine. HCV-11 was resuspended in coupling buffer (22). HCV-239 is a 4th-generation recombinant antigen and contains epitopes to HCV core, NS3, NS4, and NS5 proteins. The HBV antigen (HBV-270), an Escherichia coli-derived HBV core recombinant protein, was also purchased from ProSpec Protein Specialists and supplied in a solution containing 7.5 mM phosphate buffer (pH 7.2), 75 mM NaCl, and 50% glycerol. All antigens were stored at −70°C.

### (iv) HSV antigens.

Both the rHSV-1 and rHSV-2 antigens (recombinant gG1 and recombinant gG2) were purchased from Virusys Corp. (Taneytown, MD). The two antigens were used for the diagnosis of HSV-1 and HSV-2, respectively. Both recombinant glycoprotein G’s were derived from the host cell baculovirus (Sf-9) and then purified.

Coupled beads were vortexed and sonicated before use. A 50-μL volume containing 1,000 beads of each coupled bead region was transferred to each well in a round-bottom, 96-well polystyrene plate (Corning Life Science, Union City, CA) in the monoplex format, or a μ50-μL volume containing 10,000 beads from 10 bead regions was transferred to each well in a multiplex format. Samples were diluted, and the assay was performed as previously described (22). Additionally, internal controls were included in each plate, and the controls were selected such that in combination, the three controls were positive or negative for all pathogens in the assay. The classification of each specimen as positive or negative was based on the magnitude of the mean fluorescence intensity (MFI), using cutoffs that were established from optimization. These results were acquired from the Luminex Magpix system as previously described (22).

### Statistical analysis.

Statistical analyses were performed using GraphPad Prism analysis and Microsoft Excel. Kappa scores are categorized as follows: 0.61 to 0.8 as good, 0.81 to 0.99 as very good, and 0.99 to 1.0 as perfect. The diagnostic sensitivity and specificity were calculated using numerical values of true positives, true negatives, false negatives, and false positives (FPs) with 95% Clopper-Pearson confidence intervals (CIs). A Bland-Altman analysis was also conducted to assess how much the multiplex results are likely to differ from the monoplex results for each of the four diseases. The log of the MFIs was used in this analysis to create a normal distribution. The plot of the difference against the mean was used to determine the relationship between measurement error and the true value. Given that the true value is unknown, the mean of the two measurements was used as a proxy for the true value. Agreement between these two testing methods was determined by the mean difference and the standard deviation.

## RESULTS

### Monoplex optimization of each analyte.

Using previously determined experimental conditions, HIV antigens (1 μg of p24-gp41, 10 μg of HIV-2 IDR, and 0.04 μg of rIDR-M) were coupled to 1.5 million beads (22), while the concentrations of other analytes were systematically titrated. The optimal concentration of each antigen determined is summarized in Table 2. The optimal concentration of each antigen was the concentration that showed maximum differentiation between antibody-positive (high-MFI) and antibody-negative (low-MFI) specimens for each of the analytes. We found the optimal concentrations (for every 1.5 million beads) to be 4.5 μg for rp17, 1.5 μg for TmpA-βgal, 0.5 μg for HCV-11, 1 μg for HCV-239, 10 μg for HBV-270, and 1 μg for both rHSVgG1 and rHSVgG2. These optimal antigen concentrations were determined to maximize the sensitivity of the detection of each pathogen without severely compromising its specificity. Following the initial optimization of antigen concentrations, a pilot multiplex (10-plex) assay was performed on the combined panels (panels 1 to 5), and the results were compared with those of the monoplex runs (data not shown). All antigens in the multiplex format showed results (both qualitative and quantitative) similar to the monoplex data for positive and negative sera; hence, there was no observed cross-reactivity and/or interference.

### Reference testing of the performance evaluation panel.

All specimens in the performance evaluation panel were first characterized using standard reference tests. Hence, all 417 specimens were screened for the presence of antibodies to HIV-1, HIV-2, T. pallidum, HBV, HCV, HSV-1, and HSV-2 antigens as described above. Each panel (top row, Table 3) was used to test for the presence of pathogens using reference diagnostic tests (side, test performed, Table 3). For example, the HIV panel was used to screen for the presence of HIV, T. pallidum, HSV, HCV, and HBV. The panel tested positive for HIV-1 (*n* = 37), HIV-2 (*n* = 5), T. pallidum (*n* = 9), hepatitis B (*n* = 37), hepatitis C (*n* = 14), HSV-1 (*n* = 66), and HSV-2 (*n* = 54), for a total of 222 infections in the HIV panel alone. Seventy specimens had antibodies to HIV-1, 5 to HIV-2, 54 to T. pallidum, 78 to HBV, 67 to HCV, 283 to HSV-1, and 211 to HSV-2 antigens, for a total of 768 infections in the 417 samples (Table 3). These results were used as reference data to calculate the sensitivity and specificity of the newly developed multiplex assay, as shown in Table 4.

**TABLE 3:** Evaluation of the 10-plex assay using well-characterized specimens^*a*^

Test performed	No. of positive results with panel^*b*^					
	HIV (*n* = 85)	T. pallidum (*n* = 85)	Hepatitis (*n* = 85)	HSV (*n* = 85)	Supplemental (*n* = 77)	Total
HIV-1	37	32	0	0	1	70
HIV-2	5	0	0	0	0	5
T. pallidum	9	42	2	1	0	54
Hepatitis B	37	17	20	2	2	78
Hepatitis C	14	8	42	0	3	67
HSV-1	66	60	54	45	58	283
HSV-2	54	50	37	44	26	211

Total	222	209	155	92	90	768

a Each subpanel from the performance evaluation panel (PEP) was tested against the presence of other pathogens. For example, the syphilis panel tested positive for HIV-1 (*n* = 32), T. pallidum (*n* = 42), hepatitis B (*n* = 17), hepatitis C (*n* = 8), HSV-1 (*n* = 60), and HSV-2 (*n* = 50). PEP specimens were positive for a total of 768 infections.

b Shading in the table signifies the number of positive samples that would be normally detected by a single assay. All others not shaded in the column or row are only detected because of the ability to multiplex.

**TABLE 4:** Performance parameters of the 10-plex assay^*a*^

Analyte	Antigen(s)	No. of results					% sensitivity	95% CI of % sensitivity	% specificity	95% CI of % specificity	% agreement	95% CI of % agreement	Kappa score	95% CI of kappa score
		TP	TN	FP	FN	Sum								
HIV-1^*b*^	p24-gp41	70	347	0	0	417	100.0	94.9, 100.0	100.0	98.9, 100.0	100.0	99.1, 100.0	1.00	1.00

HIV-2^*c*^	HIV-2 IDR, rIDR-M	5	412	0	0	417	100.0	47.8, 100.0	100.0	99.1, 100.0	100.0	99.1, 100.0	1.00	1.00

T. pallidum ^*d*^	rp17-βgal	46	358	5	8	417	85.2	72.9, 93.4	98.6	96.8, 99.5	96.9	94.7, 98.3	0.88	0.79, 0.93
	TmpA-βgal	40	348	15	14	417	74.1	60.4, 85.0	95.9	93.3, 97.7	93.0	90.2, 95.3	0.69	0.59, 0.80

HBV^*e*^	HBV-270	72	331	6	6	415^*g*^	92.3	84.0, 97.1	98.2	96.2, 99.3	97.1	95.0, 98.5	0.91	0.85, 0.96

HCV^*e*^	HCV-11	48	347	1	19	415^*g*^	71.6	59.3, 82.0	99.7	98.4, 100.0	95.2	92.7, 97.0	0.80	0.71, 0.88
	HCV-239	46	338	10	21	415^*g*^	68.7	56.2, 79.4	97.1	94.8, 98.6	92.5	89.6, 94.9	0.71	0.61, 0.80

HSV-1^*f*^	rHSVgG1	273	129	5	10	417	96.5	93.6, 98.3	96.3	91.5, 98.8	96.4	94.1, 98.0	0.92	0.88, 0.96

HSV-2^*f*^	rHSVgG2	199	197	9	12	417	94.3	90.3, 97.0	95.6	91.9, 98.0	95.0	92.4, 96.9	0.90	0.86, 0.94

a Shown are parameters of antibody binding to different antigens in the Luminex 10-plex IgG assay compared to standard diagnostic tests. TP, true positive; TN, true negative; FP, false positive; FN, false negative.

b ELISAs and Western blot assays were the standard tests used for the reference data.

c The Multispot assay was the standard test used for the reference data.

d The rapid plasma reagin, TP-PA, and Trep-Sure enzyme immunoassay were the standard tests used for the reference data.

e The Vitros Diagnostics assay was the standard test used for the reference data.

f The Final Focus and Trinity Biotech assays were the standard tests used for the reference data.

g Some specimens were depleted, and the sample volume was not large enough to run the assay.

### Multiplex (10-plex) assay on the performance evaluation panel (*n* = 417).

**FIG 1:**
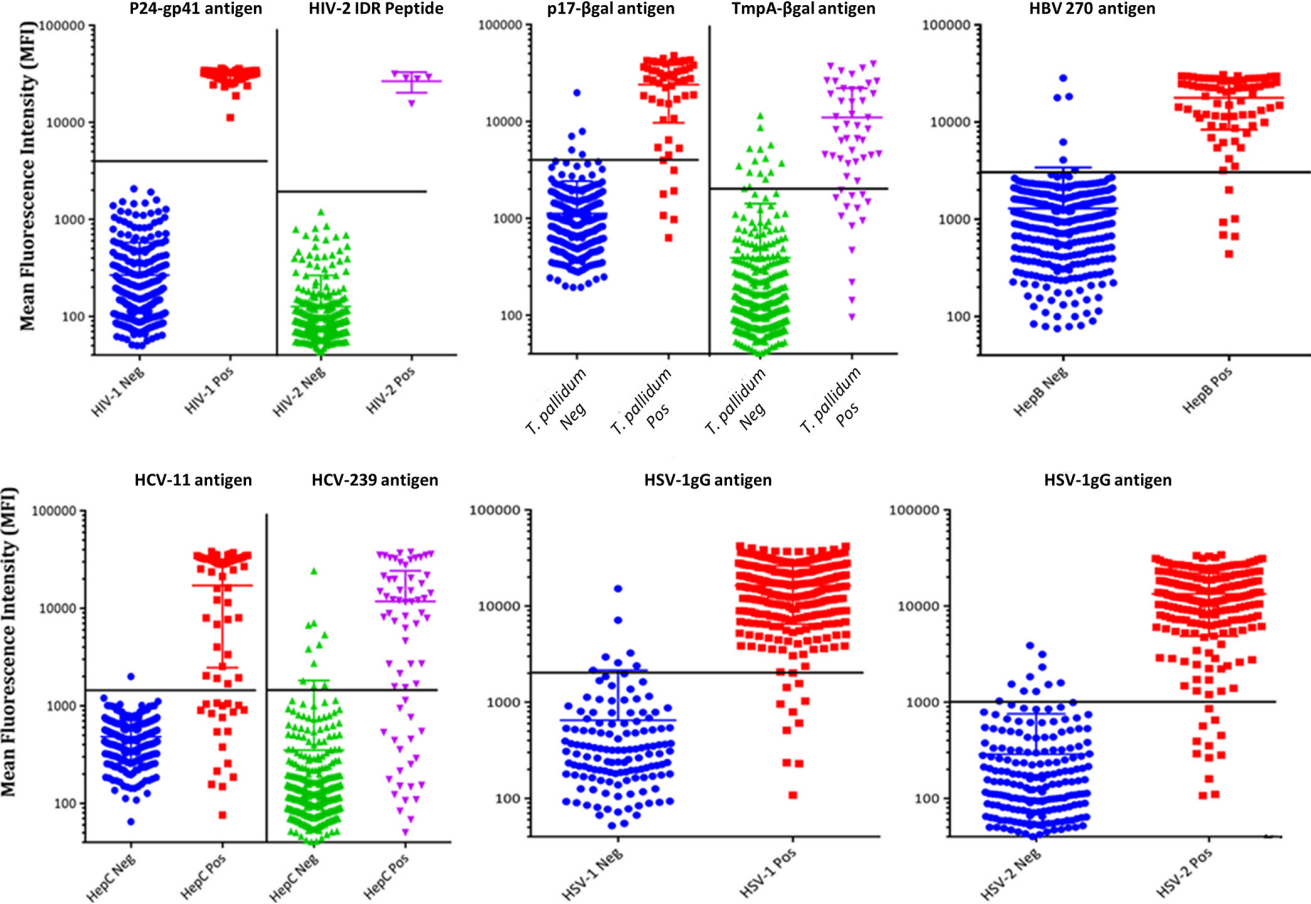
Dot plot showing the distribution and antibody reactivity levels of the 10-plex antigens. The horizontal lines (black) show the cutoffs that separate the two groups within each parameter, and the middle horizontal line within each group indicates the mean value for the group (*n* = 417). Note that background noise (MFI from beads only) was not subtracted as its contribution was minimal to the overall MFI.

**FIG 2:**
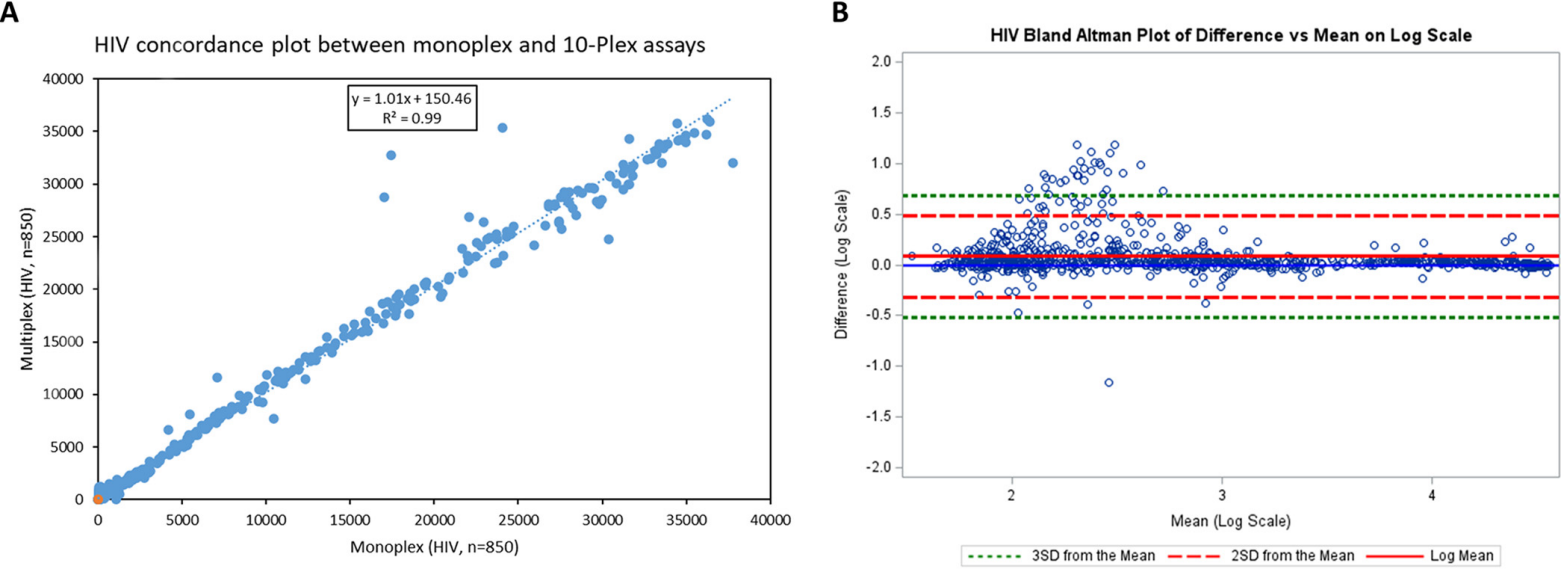
Concordance plot (A) and Bland-Altman analysis (B) between the monoplex assay and the multiplex assay. (A) MFI values were obtained from an analysis of a mixture of HIV-negative and HIV-positive specimens and analyzed for HIV reactivity in the monoplex (HIV antigens only) and multiplex (HIV antigens and all other antigens) formats. The line of best fit is represented by the dotted blue line with the statistics shown. (B) Bland-Altman plot comparing the monoplex to multiplex results on a log scale (*n* = 850).

The settings and parameters determined with the small panels (panels 1 to 5) in both the monoplex and 10-plex pilot experiments were extended to the performance evaluation panel specimens for both monoplex and 10-plex analyses. The results of the multiplex analysis are illustrated in Fig. 1 and summarized in Table 4. All analytes showed satisfactory sensitivities (HIV-1 = 100%, HIV-2 = 100%, rp17-βgal = 85.2%, TmpA-βgal = 74.1%, hepatitis B = 92.3%, HCV-11 = 71.6%, HCV-239 = 68.7%, HSV-1 = 96.5%, and HSV-2 = 94.3%) and specificities (HIV-1 = 100%, HIV-2 = 100%, rp17-βgal = 98.6%, TmpA-βgal = 95.9%, HBV = 98.2%, HCV-11 = 99.7%, HCV-239 = 97.1%, HSV-1 = 96.3%, and HSV-2 = 95.6%). Details of other parameters such as the percent accuracy and kappa coefficient are illustrated in Table 4. The overall accuracy of the 10-plex assay with the standard assays was 96.2%. There was a strong correlation between monoplex and 10-plex results as shown by the HIV subpanel analysis, with an *R*^2^ value of 0.99 and a slope of 1.01 (Fig. 2A). The results of the Bland-Altman analysis, as shown for the HIV subpanel, indicate that there is good agreement between the multiplex and monoplex results, with no systematic errors (Fig. 2B). The data are scattered relatively evenly around the mean, and the limits are narrow and reasonable. As the mean increases, there is no discernible pattern such as an upslope or starting out narrow and then widely scattering, indicating no difference (i.e., bias) and good agreement between the monoplex and multiplex data (Fig. 2B). Additionally, the correlation between the difference and the mean for HIV is very low at 0.20 (with 95% limits of agreement of −0.31, 0.47). Correlation and Bland-Altman analysis plots for other pathogens showed similar results (see Fig. S1 and S2, respectively, in the supplemental material).

## DISCUSSION

Here, we have described the development of a 10-plex assay for the simultaneous detection of IgG for antigens from HIV-1, HIV-2, HIV-1/2, T. pallidum, HBV, HCV, HSV-1, and HSV-2. The 10-plex assay offers an opportunity to detect antibodies to multiple infections simultaneously. Our 10-plex assay results agreed well with the reference data, with an overall sensitivity of 92.2% and an overall specificity of 98.1% when HCV antigens were combined for diagnosis. In this case, where multiple antigens were used to determine the diagnosis of one pathogen (hepatitis virus and T. pallidum), specimens were positive if they contained antibodies to either of the antigens. This resulted in a kappa score of 0.89. The sensitivities and specificities of each of the pathogens were also assessed at the monoplex level or intrapathogen plex level (i.e., focusing on the different antigens used within a pathogen, e.g., triplex for HIV) and at the 10-plex level. The sensitivities and specificities of the monoplex or intrapathogen plex and the 10-plex assays were identical, indicating that there was no interference or cross-reactivity from multiplexing. Examples of a monoplex and a 10-plex concordance plot are shown in Fig. 2A for HIV and Fig. S1 in the supplemental material for all other pathogens. The results for HSV, T. pallidum, HCV, and HBV were also identical. Bland-Altman plots also showed good agreement between the two tests, with no systematic errors between the monoplex and multiplex data and with a low correlation between the difference and the mean (Fig. 2B and Fig. S2).

Most of the antigens in the 10-plex assay performed well, as is the case with the HIV antigens with 100% sensitivity, followed by the HSV and HBV antigens with sensitivities of >92%. The lowest-performing antigens still had good kappa scores of 0.69 for TmpA-βgal and 0.71 for HCV-239. All other antigens had either very good kappa scores or perfect scores (Table 4). The sensitivities of rp17 (85.2%) and TmpA (74.1%) were low compared to those of all other antigens with the exception of HCV antigens. The performance of rp17 agrees with previous findings by Sambri et al., although we noted a reduced sensitivity of TmpA compared to previously reported ranges (28).

The multiplex assay correctly yielded true-negative results for 30 specimens categorized as BFPs or false positives (FPs) by the T. pallidum reference tests. BFPs represent specimens that were reactive to only RPR and nonreactive by other treponemal tests (TP-PA and Trep-Sure EIA), indicating their nonspecific reactivity due to diseases other than T. pallidum (29), whereas FPs were reactive to only treponemal EIA but were nonreactive by the RPR and the second treponemal test, TP-PA. These data suggest that the specificity of our developed multiplex system is similar to that of routine T. pallidum diagnosis. Therefore, the low sensitivity of the T. pallidum antigens can, in part, be attributed to the poor diagnostic capability of the T. pallidum standard tests. When T. pallidum testing was removed from the algorithm, the overall sensitivity improved ∼1.5% but with a marginal increase in specificity (0.3%). Comparatively, the HCV antigens did not perform well with the least sensitive antigen, HCV-239, contributing to an overall decrease in sensitivity by ∼1.5%. However, when HCV-11 and HCV-239 were combined, the sensitivity of the HCV assay increased to 79.1%, as opposed to 71.6% and 68.7%, respectively, for the individual antigens.

Most HSV and viral hepatitis specimens that were false negative by the 10-plex assay were borderline specimens by the HSV, HBV, and HCV reference tests. The likelihood of these specimens being classified differently upon retesting by the reference test is therefore very high. Most of these borderline specimens were negative by both the monoplex and the 10-plex assays and could be true negatives that were poorly characterized by the reference tests. The bead assay simulates solution-phase kinetics where the antigen and antibody are both in the liquid phase compared to reference tests where the antigen is in the solid phase and the antibody is in the liquid phase. Presumably, liquid-liquid-phase reaction kinetics have a greater tendency to mix homogeneously with decreased nonspecific binding compared to solid-liquid-phase kinetics. Coupled with the stringent buffer system and the shaking during incubations, it is tempting to posit that these positive specimens by reference tests are truly negative as suggested by the multiplex assay. It is also possible that these specimens are recent infections that have not developed high levels of antibodies. However, borderline false positives are not uncommon for even highly sensitive diagnostics tests such as 3rd- and 4th-generation enzyme immunoassays for HIV diagnosis (30–32).

The classification of HIV-1/2 dual infection and HIV-2 was aided by the rIDR-M antigen. rIDR-M is a multisubtype gp41 recombinant protein that is used in the limiting antigen avidity assay for the classification of recent and long-term HIV-1 infections (33). Owing to its design and when used in limiting concentrations, the rIDR-M protein does not bind HIV-2 antibodies. This quality of the antigen helps in serotyping of the virus. The rIDR-M antigen was used in limiting concentrations to distinguish HIV-1/2 dual infections from HIV-2 infections using an algorithm that is dependent on the binding efficiency (reflected by the MFI) of the antigen for the antibodies present in the sample. The simultaneous binding of the p24-gp41 antigen, rIDR-M protein, and HIV-2 peptide is suggestive of dual infection, while the binding of only HIV-2 peptide and, less commonly, p24-gp41 in cross-reactivity cases is suggestive of HIV-2 infection only.

The flexibility afforded by the multiplex system makes it possible to add more antigens to the detection system as desired without changing assay conditions for at least up to 20 analytes (data not shown). The Luminex Magpix platform permits up to 50 individual analytes per well. As shown, intrapathogen multiplexing also increases the sensitivity of detection by combining multiple antigens where the overall sensitivity is better than single-biomarker sensitivity. In addition to comprehensive reductions in time and cost, the multiplex assay in this format may reveal coinfections and disease patterns during surveillance that are not easily recognized. Multiplexing delineates population subgroups that are at risk of the most severe blood-borne pathogens since the presence of any of these pathogens is a proxy marker for unprotected sex or at-risk behavior.

This 10-plex multiplex assay has the potential to improve and simplify disease surveillance in resource-limited settings by testing for multiple pathogens at once. A robust disease surveillance system is important for controlling and/or eliminating the transmission of these diseases. Early diagnosis is important in preventing MTCT, and while further work is needed to optimize the 10-plex assay, this has the potential to drastically reduce the disease burden in resource-limited settings where testing has often been limited. The cost of this test remains lower than those of the single and dual tests, providing a reasonable-cost alternative while increasing access to testing. The high sensitivity and specificity of the 10-plex assay also reduce concerns over false-negative and -positive rates, which is especially important given the concerns over unnecessary further testing and medication as well as the potential emotional trauma that it can give to patients who have a false-positive result. Additionally, it is important to note that the 10-plex assay is a 96-well-plate-based assay that overcomes many of the laboratory infrastructure requirements of the individual diagnostic tests, thereby simplifying the logistics associated with testing and the acquisition of individual diagnostic testing. These factors make it a more accessible test for many resource-limited settings that may not have either the technical knowledge or the infrastructure capacity to conduct the individual diagnostic tests using a dedicated platform. While a detailed cost-comparative analysis is in progress, based on this proof-of-concept study, this has substantial potential to improve diagnostic testing. In addition to this analysis, we are also in the process of conducting several field validations for the multipathogen assay and using clinical samples from antenatal clinics.

The ability to arbitrate discordant specimens was limited due to the low volume and lack of clinical information associated with the specimens. It is also possible that the antigens used were not optimal. One of the limitations can be attributed to the absence of optimally performing reference tests for T. pallidum. Our assay uses only HBV core antigens and shows the presence of only total anti-HBc antibodies, which are a marker of exposure to HBV infection. In the absence of the detection of antibodies to hepatitis B surface antigen, the assay therefore does not distinguish between immunological responses induced by HBV infection and those induced by hepatitis B vaccination.

### Conclusion.

Despite groundbreaking advances in diagnostic technology, the diagnosis of common infections remains a challenge, especially for low-income countries. We present a proof of principle that the diagnosis of multiple pathogens can be performed using a simple assay that requires a minimal sample value and in a single run. Our results show that the assay is robust, with great accuracy and reproducibility. The ability to multiplex reduces error rates and the cost of diseases surveillance given that multiplexing, while algorithm driven, is a single test performed by one tester. The cumulative error rate is significantly decreased compared to that with a tester conducting each test individually. The simultaneous classification of a single specimen for the presence or absence of multiple pathogens in a single assay has the potential to revolutionize public health and diseases surveillance, making multiplexing the future of diseases detection. These data suggest that by using an algorithm and specific cutoffs, this multiplex assay can be used to identify multiple coinfections in addition to confirming the presence or absence of HIV-1 and HIV-2 antibodies. In conclusion, the 10-plex assay for the screening of blood-borne pathogens could help prevent transmission from mother to infant in resource-limited settings where the detection of individual pathogens can be logistically challenging and expensive. Once evaluated using specimens of maternal origin, the system would have broad applications for maternal health.
