# Supplementary material for: Development of a Bead-Based Multiplex Assay for Use in Multianalyte Screening and Surveillance of HIV, Viral Hepatitis, Syphilis, and Herpes
Source: J Clin Microbiol. 2022 Apr 7;60(5):e02348-21. doi: 10.1128/jcm.02348-21 (PMC9116187; doi:10.1128/jcm.02348-21)
Supplement: Supplemental file 1 — Fig. S1 and S2. Download jcm.02348-21-s0001.pdf, PDF file, 0.3 MB [file jcm.02348-21-s0001.pdf]

Supplemental Figures

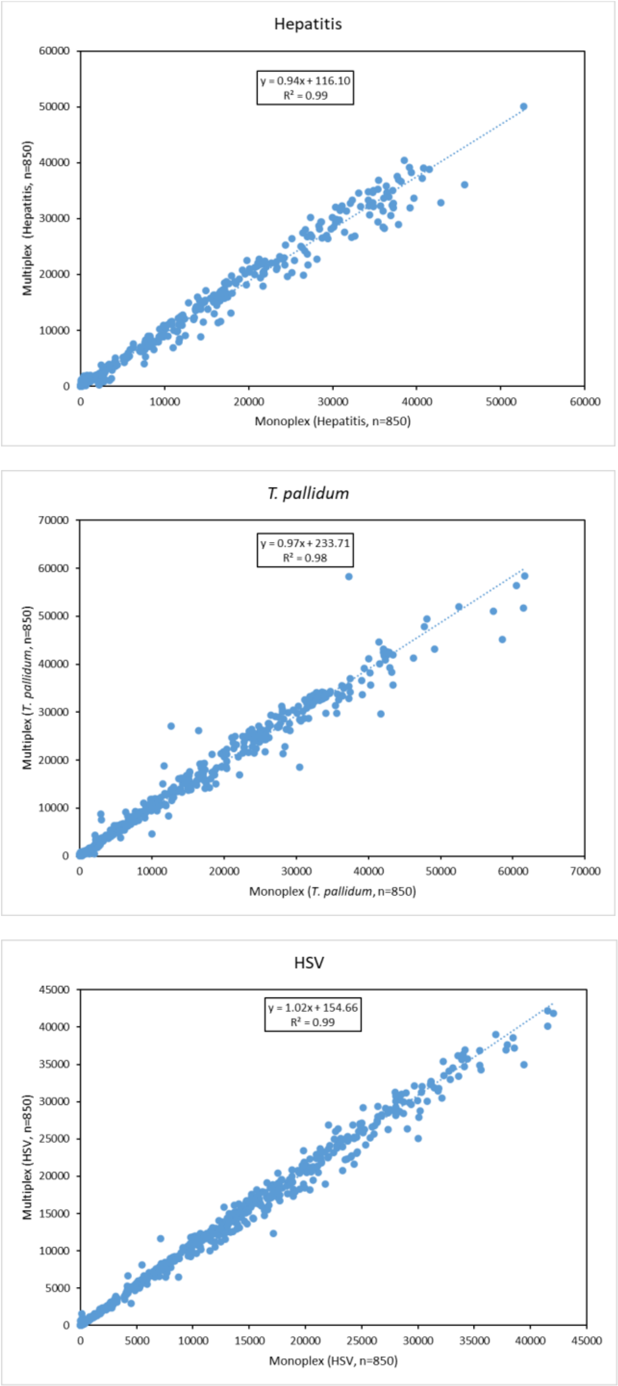

Figure S1 Concordance plot between the monoplex assay and the multiplex (10-Plex) assay. MFI values obtained from the analysis of a mixture of negative and positive specimens and analyzed for reactivity in the monoplex and multiplex formats for the various pathogens indicated. Line of best fit is represented by the dotted blue line with statistics shown.

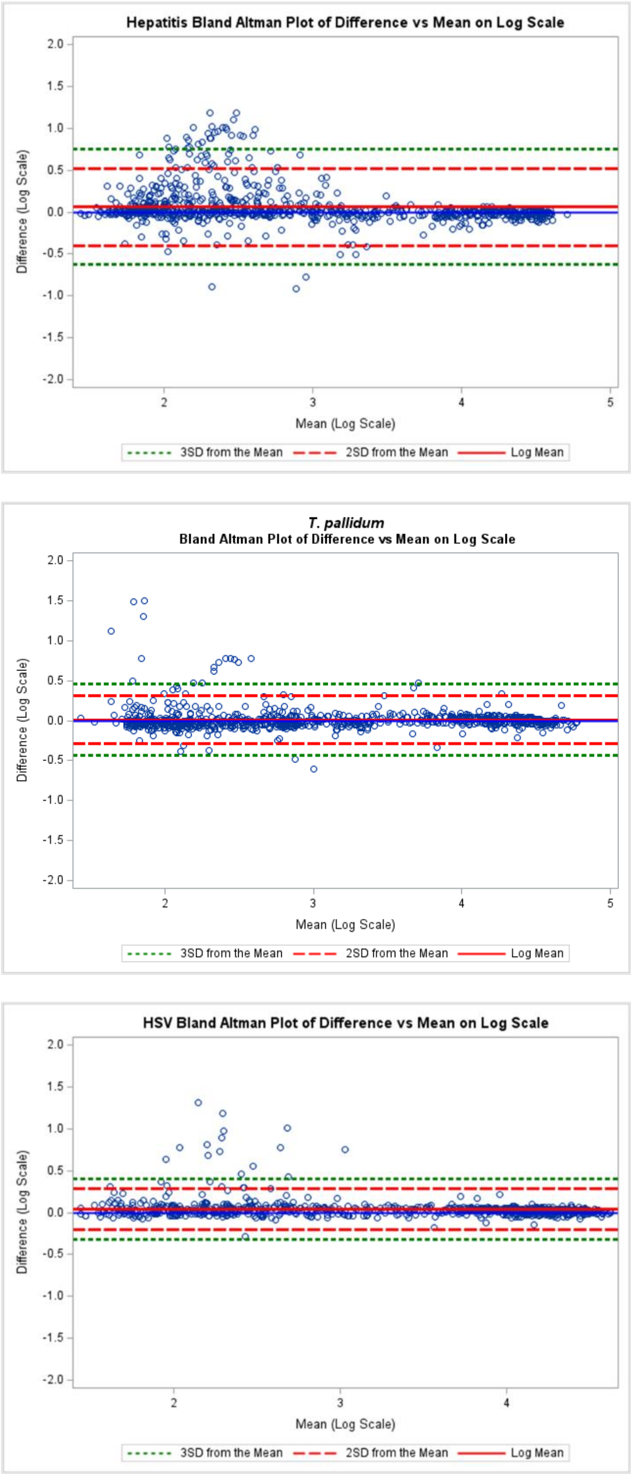

Figure S2 Bland Altman analysis Plot of Difference vs Mean on Log scale comparing monoplex and multiplex (10-Plex) results of the pathogens indicated. Statistical details are shown as standard deviations from the mean.
